# Sunlight-Driven Photocatalytic Degradation of Methylene Blue with Facile One-Step Synthesized Cu-Cu_2_O-Cu_3_N Nanoparticle Mixtures

**DOI:** 10.3390/nano13081311

**Published:** 2023-04-08

**Authors:** Patricio Paredes, Erwan Rauwel, David S. Wragg, Laetitia Rapenne, Elias Estephan, Olga Volobujeva, Protima Rauwel

**Affiliations:** 1 Institute of Forestry and Engineering Sciences, Estonian University of Life Sciences, Kreutzwaldi 56/1, 51014 Tartu, Estonia; patricio.paredes@emu.ee (P.P.); erwan.rauwel@emu.ee (E.R.); 2 Department of Chemistry and SMN, University of Oslo, 0315 Oslo, Norway; d.s.wragg@smn.uio.no; 3 Grenoble Institute of Engineering, LMGP, University Grenoble Alpes, CNRS, F-38000 Grenoble, France; laetitia.rapenne@grenoble-inp.fr; 4 Laboratory of Bioengineering and Biosciences, LBN, Univ Montpellier, 34193 Montpellier, France; 5 Institute of Materials and Environmental Technology, Tallinn University of Technology, 19086 Tallinn, Estonia; olga.volobujeva@taltech.ee

**Keywords:** photocatalysis, nanoparticles, sunlight, Cu_3_N, dye degradation, semiconductor band bending

## Abstract

Sunlight-driven photocatalytic degradation is an effective and eco-friendly technology for the removal of organic pollutants from contaminated water. Herein, we describe the one-step synthesis of Cu-Cu_2_O-Cu_3_N nanoparticle mixtures using a novel non-aqueous, sol-gel route and their application in the solar-driven photocatalytic degradation of methylene blue. The crystalline structure and morphology were investigated with XRD, SEM and TEM. The optical properties of the as-prepared photocatalysts were investigated with Raman, FTIR, UV-Vis and photoluminescence spectroscopies. The influence of the phase proportions of Cu, Cu_2_O and Cu_3_N in the nanoparticle mixtures on the photocatalytic activity was also investigated. Overall, the sample containing the highest quantity of Cu_3_N exhibits the highest photocatalytic degradation efficiency (95%). This enhancement is attributed to factors such as absorption range broadening, increased specific surface of the photocatalysts and the downward band bending in the p-type semiconductors, i.e., Cu_3_N and Cu_2_O. Two different catalytic dosages were studied, i.e., 5 mg and 10 mg. The higher catalytic dosage exhibited lower photocatalytic degradation efficiency owing to the increase in the turbidity of the solution.

## 1. Introduction

Presently, wastewater treatment is one of the most critical issues, owing to the release of large amounts of industrial effluents into water bodies [1]. In that regard, the use of organic dyes in food, papermaking, cosmetics, pharmaceuticals and textiles poses a threat to the environment [2]. Presently, different aqueous remediation methods against organic pollutants are being used, including advanced oxidation processes [3]. Today, heterogeneous photocatalysis is considered an efficient and environmentally friendly method for the degradation or removal of water-soluble organic pollutants [4,5]. To that end, several nanomaterials based on oxides and nitrides have been synthesized and studied in order to enhance the degradation of aqueous contaminants using photocatalytic processes. In particular, Cu-based nanomaterials have attracted interest due to the earth abundance of Cu, which allows for tackling issues related to the sustainability and cost-effectiveness of photocatalytic processes [6,7]. Therefore, cuprous oxide (Cu_2_O) and copper nitride (Cu_3_N) semiconductors, with narrow band gaps of 1.2–2.5 eV for Cu_2_O [8,9,10] and 0.2–2 eV for Cu_3_N [11], are seen as promising visible-light-activated photocatalysts.

In recent years, scientists have provided some insights into the photocatalytic degradation mechanism of organic dyes using Cu-based nanomaterials. Norzaee et al. reported a 90% degradation efficiency of aniline after 90 min under a UV-C lamp with CuO nanoparticles [12]. Yu et al. synthesized Cu_2_O nanoparticles using the liquid-phase reduction method, which degraded 83.2% of fluroxypyr under a 500 W metal halide lamp [13]. Sithole et al. reported a photocatalytic degradation of 61% for methylene blue and 89% for methylene orange by Cu_3_N nanocubes after 180 min and 240 min, respectively, using a solar simulator [14]. Furthermore, metallic Cu nanoparticles produce free-electron resonance under visible light excitation and are therefore potential photocatalysts. For instance, Zhang et al. demonstrated that the surface plasmon resonance (SPR) of Cu nanoparticles engenders a spontaneous photocatalytic water-splitting [15]. In fact, plasmonic metal nanoparticles combined with other luminescent nanoparticles tend to augment the overall visible absorption range and also act as a sink for excited electrons that, in turn, reduces excitonic recombination [16,17]. Even though there are some reports on the semiconductor-metal-based photocatalyst-heterostructures [18], to the best of our knowledge, the synthesis of Cu-Cu_2_O-Cu_3_N nanoparticle mixtures applied to photocatalytic dye degradation has not yet been reported.

In the present work, a novel synthesis route that allows varying the proportions of the crystalline phases of Cu, Cu_2_O and Cu_3_N using a non-aqueous sol-gel method was devised. The samples were characterized with powder X-ray diffraction (PXRD), scanning electron microscopy (SEM), transmission electron microscopy (TEM), Fourier transform infrared (FTIR), Raman, UV-Vis and photoluminescence spectroscopies. The objective is to understand the influence of the nanoparticle proportions and the catalyst dosage on the photocatalytic properties. Herein, the photocatalytic performances of the samples in degrading methylene blue (MB) dye under solar radiation was evaluated using a commercial hand-held Lovibond photometer, which also allows on-site analysis of dye-contaminated aqueous media in real-life situations.

## 2. Materials and Methods

### 2.1. Materials

Copper (II) nitride trihydrate 99% (Cu(NO_3_)_2_·3H_2_O) was purchased from Acros Organics; 1-octadecene (90%), oleylamine (90%) and MB were purchased from Thermo Scientific; and 2-propanol was purchased from Honeywell. All chemicals were of analytical grade and used as received without further purification.

### 2.2. Synthesis of Cu-Based Nanocomposite

The Cu-Cu_2_O-Cu_3_N nanoparticle mixtures were synthesized using a non-aqueous sol-gel route inside a glovebox (controlled N_2_ atmosphere), and the reagents were sealed in an autoclave for the synthesis [19,20]. In a typical experiment, 0.3 g of Cu(NO_3_)_2_·3H_2_O was dissolved in 10 mL of Octadecene. Then, 10 mL of oleylamine was added and magnetically stirred at 110 °C for 20 min until a homogenous solution was obtained. Subsequently, the solution was placed inside an autoclave and heated in an oven at different reaction intervals of 3, 6, 12 and 24 h at a temperature of 280 °C, and the samples were named Cu-3, Cu-6, Cu-12 and Cu-24, respectively. The autoclave was then removed from the oven, and the reaction mixture was allowed to cool down to room temperature. Finally, the precipitated nanomaterials were isolated using centrifugation, washed twice with isopropanol and dried.

### 2.3. Characterization

The crystalline phase, proportions and particle sizes were examined with PXRD using a Bruker D8 Discover diffractometer (Bruker AXS, Karlsruhe, Germany) with CuKα1 radiation (λ = 0.15, 406 nm) selected with a Ge (111) monochromator and LynxEye detector. Phases were identified using the crystallography open database [21] in Bruker EVA version 6.0. Diffraction patterns were fitted using the Rietveld method in TOPAS version 6 [22] to obtain phase weight percentages and crystallite sizes (Scherrer method with full profile fit, k = 0.89). The peak shape was modeled using a fundamental parameters approach and the cubic lattice parameter, scale and Lorentzian size broadening were refined for three phases: copper (I) nitride (COD 1010167), cuprite (COD 1000063) and metallic copper (COD 9013014). The microstructure of the samples was characterized with the use of transmission electron microscope (TEM) JEOL 2010 LaB_6_ TEM (JEOL, Japan) operating at 200 kV in TEM mode and providing a point-to-point resolution of 1.9 Å. The surface morphology was investigated with a high-resolution scanning electron microscope HR-SEM Zeiss Merlin (Carl Zeiss Microscopy, Munich, Germany), and the chemical composition was determined using an energy dispersive X-ray analysis (EDS) system Bruker EDX-XFlash6/30 detector (Bruker, Oxford, UK) with an acceleration voltage of 4 kV for SEM and 10 kV for EDX analysis. The elemental composition quantification was performed using PB/ZAF standard less mode. Another SEM, EVO LS15 Zeiss (Carl Zeiss Microscopy, Germany), working in backscattered and secondary electron mode at a voltage of 15 kV was used to study the morphology and topographic characteristics of the Cu-based nanocomposites. The vibrational properties were studied using a WITec Confocal Raman Microscope System alpha 300R (WITec Inc., Ulm, Germany). Excitation in confocal Raman microscopy is generated with a frequency-doubled Nd:YAG laser (New-port, Irvine, CA, USA) at a wavelength of 532 nm, with 50 mW maximum laser output power in a single longitudinal mode. The incident laser beam is focused onto the sample with a 60× NIKON having a numerical aperture of 1.0 with a working distance of 2.8 mm (Nikon, Tokyo, Japan). The acquisition time of a single spectrum was set to 0.5 s. The chemical bonds were studied using a Fourier transform infrared (FTIR) spectrometer (Nicolet is10 Thermo Scientific, Driesch, Germany) in the range of 360–1100 cm^−1^. The optical absorbance of the nanocomposites was determined using a VWR UV-VIS spectrometer (UV-1600PC, USA) in the range of 350–1100 nm. Finally, photoluminescence spectroscopy was carried out at room temperature on the nanopowders with excitation wavelengths of 365 nm and 532 nm using diode lasers LSM-365A and LSM-533A LED (Ocean insight, Orlando, FL, USA) with specified output powers of 10 mW and 1.96 mW, respectively. The emission was collected using a FLAME ES UV-Vis spectrometer (Ocean optics, USA) with a spectral resolution of 1.34 nm.

### 2.4. Adsorption Experiments

The calibration curve of MB using the Lambert–Beers law is available in Figure S1, which was obtained with a Lovibond MD 610 photometer at the MB characteristic absorption peak of 660 nm. The MB adsorption study on the photocatalysts was carried out at room temperature under dark conditions. For this, 5 mg of the absorbent was added to 15 mL of a MB (5 mg/L) stock solution. Aliquots were carefully obtained using a pipet for analysis. The amount of MB adsorbed was calculated using Equation (1):

(1) $$Q_{t}=\frac{{V(C}_{o}{\;-\;C}_{e})}{m}\;(\frac{\mathrm{mg}}{g}),$$

where Q_t_ (mg/g) is the amount of dye (MB) adsorbed/unit weight of the sample; C_0_—initial concentration of MB (mg/L); C_e_—concentration of MB at equilibrium time (mg/L); V—volume of solution (L) and m—the weight of the sample (mg). The percentage of MB adsorbed is given by R (%) as is given in Equation (2):

(2) $$R=\frac{{(C}_{0}{\;-\;C}_{t})}{m}\;100\%,$$

where C_0_ and C_t_ are the initial and final concentrations of MB in the solution, respectively.

The MB adsorption behavior of the nanoparticle mixtures was also verified for the pseudo-second-order kinetic model, which assumes that the rate-limiting step is the interaction between two reagent particles. The equation of this model is illustrated as Equation (3):

(3) $$\frac{t}{Q_{t}}=\frac{1}{k_{2}Q_{e}^{2}}+\frac{t}{Q_{e}},$$

where k_2_ is the equilibrium rate constant of the pseudo-second-order (g/mg min). The linear fit between the t/Q_t_ and contact time (t) can be approximated as pseudo-second-order kinetics.

### 2.5. Photoctalytic Experiments

The photocatalytic activity of the Cu-Cu_2_O-Cu_3_N nanoparticle mixtures was evaluated by monitoring the photodegradation of MB under solar radiation in the open air. In a typical experiment, 5 mg and 10 mg of the photocatalyst were dispersed in 20 mL of the MB aqueous solution with a concentration of 5 mg/L. For evaluating the photocatalytic activity after adsorption, the suspension was placed in the dark for 2 h in order to attain adsorption–desorption equilibrium before being exposed to solar radiation in beakers with and without a glass cover. The glass cover served as a filter to block UV radiation from the sun and allowed us to solely evaluate the visible-light, solar-driven photocatalysis.

## 3. Results and Discussion

### 3.1. Structure and Morphology

**Figure 1:**
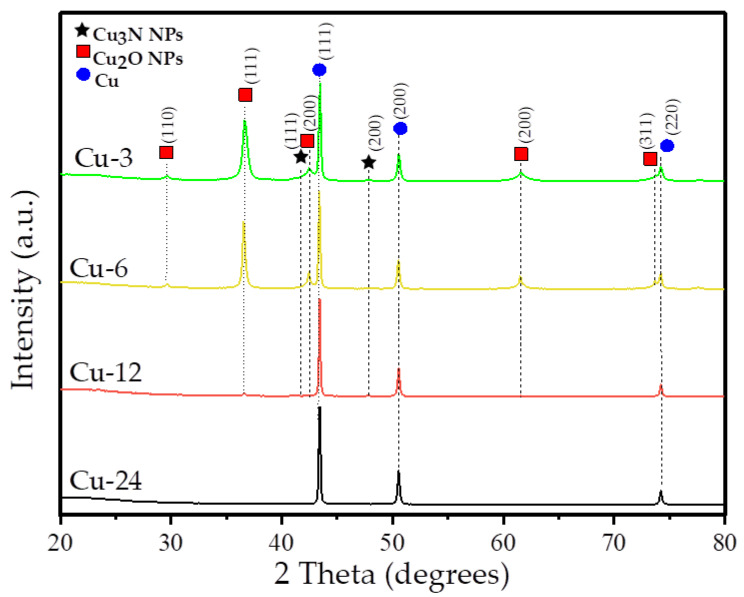
PXRD patterns of the samples.

The variation in the crystalline phase proportions of Cu_2_O, Cu_3_N and Cu in the samples as a function of synthesis time was studied using PXRD analysis (see Figure 1). The PXRD patterns of samples Cu-3, Cu-6 and Cu-12 exhibit the characteristic PXRD peaks of Cu_2_O, Cu_3_N and metallic Cu structures in variable proportions (see Table 1). This result clearly suggests that the Cu^I^ species were generated with the reduction of Cu^II^ by oleylamine upon heating in reductive environments. In the PXRD pattern, peaks at 29.68°, 36.47°, 42.3°, 61.4° and 73.6°, correspond to (110), (111), (200), (220) and (311), respectively reflections of Cu_2_Obelonging to the cubic Pn$\bar{3}$m phase (COD 1000063). The diffraction peaks visible at 41.12° and 47.84° correspond to the crystal planes (111) and (200), respectively, of Cu_3_N nanocrystals belonging to the Pm$\bar{3}$m space group (COD 1010167). The proportion of Cu metal nanoparticles increases with the synthesis time, and sample Cu-24 consists of pure metallic Cu nanoparticles, indicating a complete reduction of Cu^I^ to Cu^0^. The PXRD peaks visible at 43.26°, 50.42°and 74.12° correspond to the (111), (200) and (220), respectively, planes of Cu-metal face-centered cubic crystal structure Fm$\bar{3}$m (COD 9013014). The differences in phase composition are due to changes in the synthesis reactions, influenced by the synthesis time. In fact, as the synthesis time increases, a large decomposition or/and desorption of the oleylamine capping agent is likely, leading to the further reduction of Cu_2_O and Cu_3_N into Cu metal [23]. The PXRD data indicate that the thermal decomposition of Cu(NO_3_)_2_·3H_2_O in octadecene and olyelamine at 280 °C results in the precipitation of Cu-Cu_2_O-Cu_3_N after 3 h. Then, as the synthesis time increases, the Cu_3_N and Cu_2_O phases are reduced to Cu metal.

The crystallite size and weight percentages of Cu_2_O, Cu_3_N and Cu nanoparticles in the samples are calculated from Rietveld fits of the PXRD data. Table 1 reports the average crystallite diameter of each phase and the weight % composition of the samples from Rietveld fits to their PXRD patterns. The calculations indicate that for Cu-3, the particle sizes of Cu_2_O and Cu_3_N are 15 nm and 3 nm, respectively. As the reaction time increases for Cu-6 and Cu-12, the nanoparticles adopt an average size of 30 nm for both the Cu_2_O and Cu_3_N phases. After 24 h (Cu-24), only the diffraction peaks characteristic of Cu metal are observed. The Cu nanoparticles have an average size of 50 nm in samples Cu-3, Cu-6 and Cu-12, but for Cu-24, in which it is the only phase present, the average particle size increases slightly to 70 nm.

**Figure 2:**
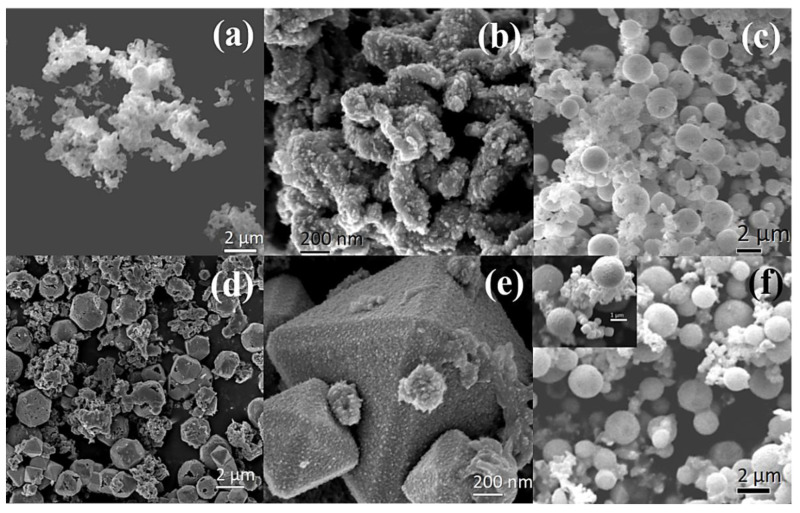
SEM images of (**a**,**b**) Cu-24, (**c**) Cu-3, (**d**) and (**e**) Cu-6, (**f**) Cu-12 (the inset is a higher magnification image of all the 3 morphologies, i.e., powder, sphere and cube).

**Figure 3:**
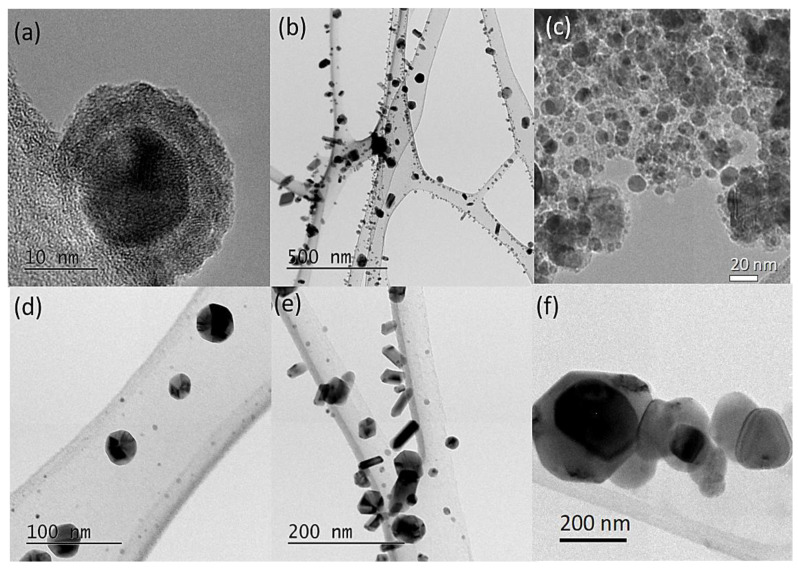
TEM images of the Cu_2_O, Cu_3_N and Cu nanoparticle mixture samples synthesized at different reaction times (**a**,**d**) Cu-3, (**b**,**e**) Cu-6, (**c**) Cu-12 and (**f**) Cu-24.

Figure 2 and Figure 3 are the typical SEM and TEM images of the samples. In Figure 2a,b, corresponding to the Cu-24 sample, the pure Cu nanoparticles exhibit a homogeneous powder morphology. On the other hand, Figure 2c, showing the Cu-3 sample, contains a blend of all three phases. However, two main morphologies are visible, including the powder morphology of the Cu nanoparticles in Figure 2a,b. The existence of sphere-like nanoparticles obtained after 3 h of synthesis can be attributed to Cu_2_O nanoparticles, considering their phase proportion of ~50%. These spheres are agglomerates of smaller nanoparticles, given their granular surfaces. For this sample, the Cu_3_N nanoparticles are not discernable because of their small average particle size of 7 nm, according to the PXRD results. With an increase in synthesis time, i.e., for the Cu-6 and Cu-12 samples (Figure 2d–f), additional morphologies are visible. Some reports suggest that the thermal decomposition of oleylamine depends on the synthesis time, which plays an important role in controlling the size and shape of the nanocrystals [24,25]. Thus, in addition to the spherical and powder morphologies corresponding mostly to Cu_2_O and Cu nanoparticles, tetrahedral and cubic morphologies are also observed. In fact, both Cu_2_O and Cu_3_N can present cube and pyramidal morphologies. In the case of Cu_2_O, several morphologies, i.e., cube, truncated octahedron and tetrapods are possible with varying the quantity of ammonia, hydroxyl groups and water in the solution [26]. In our synthesis, the quantity of ammonia in the reaction mixture under solvothermal conditions, along with the optimum reaction time, determines not only the quantity of Cu_3_N and Cu_2_O nanoparticles precipitated but also their morphology [24].

Furthermore, EDX was performed on several particles for the Cu-6 sample, in order to distinguish Cu_3_N from Cu_2_O. It should be noted that EDX is not suitable to quantify lighter elements, such as oxygen and nitrogen. However, variations in the EDX peak intensities of N and O from various particles could provide an indication of N/O-rich or poor compositions. As seen in the EDX spectrum in Figure S2, the truncated octahedral particles manifest a relatively intense oxygen peak compared to the nitrogen peak and are, therefore, most likely Cu_2_O.

TEM was carried out in order to study the particle sizes and morphologies of the Cu-based nanoparticles. It is noteworthy that the TEM images on their own are not sufficient to determine the nanoparticle phase. However, techniques such as XRD coupled with Rietveld refinement provide the average crystallite size for each phase. The size of nanoparticles from TEM was compared with PXRD data from Table 1 in order to estimate the phase of the nanoparticles. Figure 3a,d shows the micrographs of the Cu-3h sample. In Figure 3a, a nanoparticle of size 15 nm is most likely Cu_2_O, in agreement with the XRD results. However, a shell is visible on its surface (Figure 3a), probably due to the incomplete reaction of oleylamine. Several studies have shown that the presence of oleylamine surfactant modifies the optical properties of the materials. For instance, ZnO/ZnCdSe alloy synthesized with oleylamine manifested a blueshift in the absorption spectrum and a red shift in the emission spectrum [27]. A similar trend was also observed for CuO nanoparticles synthesized with oleylamine [28]. In Figure 3d, the contrast of the TEM images reveals the presence of twin boundaries, which are typical of metallic nanoparticles such as Cu with a particle size of ~50 nm. In addition, there are smaller nanoparticles in the background of ~5 nm in size, as shown in Figure 3d, which are most likely Cu_3_N (Table 1).

As the reaction time increases, changes in particle sizes and morphologies are visible. Additionally, the capping agent that was present in the Cu-3 sample is absent at longer synthesis intervals. In Figure 3b,e, the TEM images of the Cu-6 sample reveal different morphologies for Cu_2_O, Cu_3_N and Cu nanoparticles. The cuboidal nanoparticles would most probably correspond to Cu_2_O, as the crystalline proportion of Cu_3_N is very low (~1.1%). For the Cu-12 sample (Figure 3c), the TEM micrograph reveals spherical nanoparticles of ~50 nm that correspond to the Cu phase, whereas smaller nanoparticles of ~20 nm could be both Cu_3_N and Cu_2_O. Finally, the TEM image of the Cu-24 sample in Figure 3f displays pure Cu nanoparticles with sizes ranging from 50 nm to 300 nm.

### 3.2. Elemental Characterization

**Figure 4:**
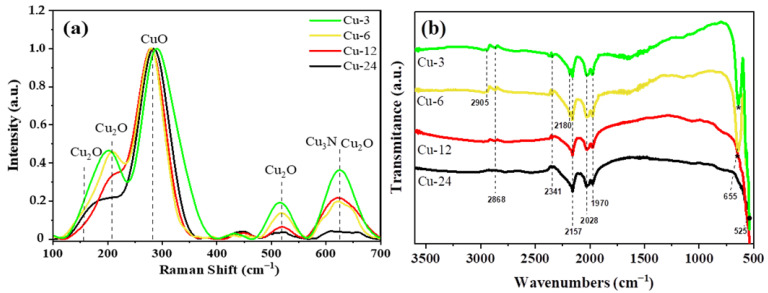
(**a**) Normalized Raman spectra and (**b**) FTIR spectra of the mixtures of the nanocomposites.

The relationship between the physical and chemical properties of the nanocomposites was probed using Raman spectroscopy, as shown in Figure 4a. In principle, the presence of Cu nanoparticles cannot be ascertained with Raman spectroscopy as metals possess negative-real and positive-imaginary dielectric constants and also exhibit surface plasmon resonance [29]. However, in the present case, Cu metal nanoparticles from the Cu-24 sample oxidized under the green laser beam into cupric oxide (CuO) [29]. The peak at 280 cm^−1^ is assigned in the literature to the stretching vibrational mode of CuO. This peak is present in all the samples due to the presence of Cu nanoparticles that systematically undergo oxidation under the laser. It should be noted that CuO is not present in the as-synthesized nanocomposites (Figure 1). However, the small redshift in this peak and the broadening in the bandwidth at around 290 cm^−1^ for the Cu-3 sample could be attributed to the small nanoparticles in the Cu_3_N phase (~3 nm). The Raman peaks at around 152 cm^−1^, 216 cm^−1^ and 515 cm^−1^ are fully consistent with peaks of Cu_2_O [30]. According to Wei et al., the peak at about 216 cm^−1^ could be attributed to the second-order overtones 2Γ_12_, and the other weaker peaks at 152 cm^−1^, 515 cm^−1^ and 624 cm^−1^ correspond to Γ_15_ of oxygen vacancies; the fourth-order overtone 4Γ_12_ and the red-allowed mode Γ_15_ are phonon vibrations of Cu_2_O [31]. The relative intensity of the peak at 515 cm^−1^ decreases with the synthesis time due to the decreasing quantity of Cu_2_O. According to Sajeev et al., the peak located at 624 cm^−1^ corresponds to the stretching and bending in the Cu-N bond from the Cu_3_N phase [32]. The relative intensities of this peak for Cu-6 and Cu-12 are almost the same due to the low amounts of Cu_3_N present in these samples. However, for the Cu-3 sample, there is an increase in the relative intensity of this peak owing to a higher amount of the Cu_3_N phase (18%). In addition, the band between 151 and 171 cm^−1^ corresponds also to the Cu-Cu dimer, which is prominent in the Cu-24 sample [33].

The FT-IR spectra, as shown in Figure 4b, highlight the vibrational bands of the organic and inorganic moieties present in the nanocomposites. FT-IR indicates changes in the framework configurations of the nanocomposites via shifts in the bands as a function of synthesis time. In the FT-IR spectra of the Cu-3 and Cu-6 samples, the peak located at 2868 cm^−1^ corresponds to the stretching vibrational mode of the Cu-O bonds from the Cu_2_O crystalline phase [34,35]. Moreover, a decrease in the peak intensity signifies a decrease in Cu_2_O content in the samples. This peak is no longer present in samples synthesized at longer synthesis intervals. Furthermore, the characteristic peak located at 655 cm^−1^ in the Cu-3 and Cu-6 samples can also be ascribed to the intrinsic lattice mode vibration in Cu-N bonds from the Cu_3_N nanoparticles present in the nanocomposites [32]. However, for the Cu-12 sample, the peak at 655 cm^−1^ is not visible due to a very low fraction in the Cu_3_N phase (1.1%), as well as for the Cu-24 sample that corresponds to pure Cu-metal nanoparticles. The main stretching vibration in pure Cu nanoparticles was found at 520 cm^−1^ (represented by •) [36]. The FTIR spectra of the Cu-3 and Cu-6 samples also exhibit a small peak at ~2905 cm^−1^ that can be attributed to the stretching vibration in the C-H bonds from the oleylamine [37]. Free N_2_ has a N≡N stretching at around 2331 cm^−1^ [38]; in contrast, because N_2_ coordinated with a metal atom is a weak Lewis base, this coordination shifts the stretching peak to 1970−2180 cm^−1^ [38].

### 3.3. Optical Properties

**Figure 5:**
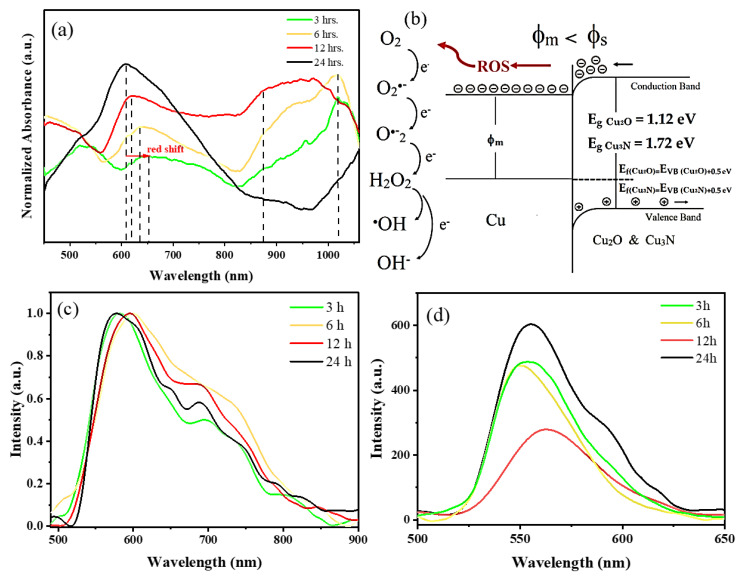
(**a**) Normalized UV-Vis absorption spectra of the nanoparticle mixtures, (**b**) downward band bending in p-type semiconductors, along with electron accumulation and transfer to the oxygen molecules generating ROS. The bandgaps of Cu_3_N and Cu_2_O were determined using the Tauc plots in Figure S4. Their band edges were then determined using the energy levels in references [49,50]. Photoluminescence emission spectra of the samples with an excitation wavelength of (**c**) 365 nm (normalized emission) and (**d**) 533 nm.

UV-Vis absorption spectra of the nanocomposites show a broad absorption peak located at ~600 nm that can be assigned to the SPR band of Cu nanoparticles (Figure 5a) [39]. This peak is prominent in the Cu-24 sample, which corresponds to a pure phase of Cu metal. For samples synthesized at shorter intervals, the SPR of the Cu-metal phase shows a redshift. It is reported that the SPR band of Cu-metal nanoparticles shifts as a function of the Cu-nanoparticle size, shape, dielectric properties and the presence of other phases in the sample [40,41,42]. For instance, Mott et al. reported a redshift in the SPR of Cu as the particle size increases [39]. In our study, the red shift in the SPR of Cu is attributed to the presence of the Cu_2_O and Cu_3_N phases, with additional absorption wavelengths in the red part of the visible spectrum and the near-infrared, respectively. For the samples that contain a higher amount of Cu_3_N nanoparticles, i.e., Cu-3 and Cu-6, a characteristic absorption peak is located at around 1020 nm, but for the Cu-12 sample, this peak is around 980 nm. These corroborate with the reported band gap of ~1.2 eV for Cu_3_N [43,44]. The absorption peaks visible at around 870 nm (1.4 eV) in the Cu-3, Cu-6 and Cu-12 samples correspond to the absorption edges of Cu_2_O nanoparticles, corroborating with the range of the reported bandgap, i.e., 1.2 eV–2 eV [10,45].

The emission properties of the nanocomposites were examined using room temperature photoluminescence spectroscopy. Under UV-excitation of 365 nm (Figure 5c), the emission peak maximum of all samples is between 580 nm and 620 nm, corroborating with the plasmonic emission of Cu-metal nanoparticles. The fluorescence is ascribed to the radiative recombination of electrons in the s-p conduction band below the Fermi level with the holes in the d-band of Cu. In addition, the asymmetrical emission spectra have a low energy tail related to intraband transitions [46]. Changes in the intensity of the intraband emission are a result of changes in the coupling of Cu with various nanocomposites, owing to electron transfer mechanisms.

Since we performed solar-driven photocatalysis, and the maximum intensity of the solar radiation is around 550 nm, we therefore chose a 533 nm excitation source to study the emission properties of the samples (Figure 5d). The photoluminescence emission spectra of Cu and Cu_2_O have been adequately studied in the literature [47]. However, very few reports exist on the photoluminescence emission of Cu_3_N using a visible light excitation source. In fact, in most of the studies, a UV excitation source with very high emission intensity was used. Yeshchenko et al. studied photoluminescence emission localized at 660 nm in Cu metal nanoparticles with an excitation wavelength of 355 nm [46]. Basavalingaiah et al. reported the photoluminescence emission of Cu_2_O with an excitation wavelength of 250 nm, showing two-emission peaks at 712 nm and 564 nm [48]. Sithole et al. studied the photoluminescence spectra of Cu_3_N nanoparticles with an emission around 486 nm using an excitation wavelength of 200 nm [14].

In the photoluminescence spectra shown in Figure 5c,d, the emission is dominated by the Cu plasmonic emission for all the samples irrespective of the presence of other phases. PL emissions corresponding to Cu_3_N and Cu_2_O were once again not observed. For the Cu-24 sample, the pure Cu metal nanoparticles exhibit a strong emission peak at 554 nm, very close to the photoluminescence band of bulk cooper (560 nm) [46]. Nevertheless, a decrease in the emission intensity along with energy shifts are also notable in the spectra, influenced by the presence of secondary phases and differences in nanoparticle sizes. According to the literature, the blueshift in the photoluminescence spectrum of the Cu-based nanoparticles is indicative of the presence of surface traps, while a redshift towards the band edge emission indicates defect-free nanoparticles [14]. The changes in the emission peak positions and intensities can be attributed to electron transfer mechanisms between various phases and changes in radiative recombination influenced by the ambient as well as semiconductor band bending. In fact, band bending is a phenomenon that occurs at the interface of a semiconductor and a metal. The nature of the band bending depends on the work functions of the two materials. In our case, the work function of Cu (4 eV) is lower than the work functions of Cu_3_N (5.06 eV) and Cu_2_O (5 eV) [51,52]. Therefore, a downward band bending in the p-type Cu_2_O and Cu_3_N occurs due to the formation of a Schottky junction with Cu nanoparticles [53,54,55,56]. As a consequence, the downward band bending reduces the radiative recombination in Cu_3_N and Cu_2_O in the band bending region (Figure 5b). Upon photoexcitation, the band bending leads to an accumulation of electrons on the surface of Cu_3_N and Cu_2_O that are subsequently transferred to Cu nanoparticles. At the same time, holes are directed toward the volume of the nanoparticle from the band-bending region. Consequently, the excitonic recombination is suppressed in Cu_3_N and Cu_2_O. On the other hand, owing to the electron transfer to Cu nanoparticles, the emission of Cu metal nanoparticles is enhanced. Simultaneously, electrons on the surfaces of Cu_2_O and Cu_3_N would also undergo non-radiative recombination, e.g., via transfer to chemisorbed oxygen molecules, when photoexcited in ambient conditions. The highest emission is observed in the Cu-24 sample containing pure Cu metal nanoparticles, while the lowest is attributed to the Cu-12 sample with Cu and Cu_2_O phases. For photocatalysis, a low emission or longer excitonic recombination lifetime is important in order to ensure electron transfer from the nanoparticle to the dye solution [54,56].

## 4. Adsorption Kinetics

**Figure 6:**
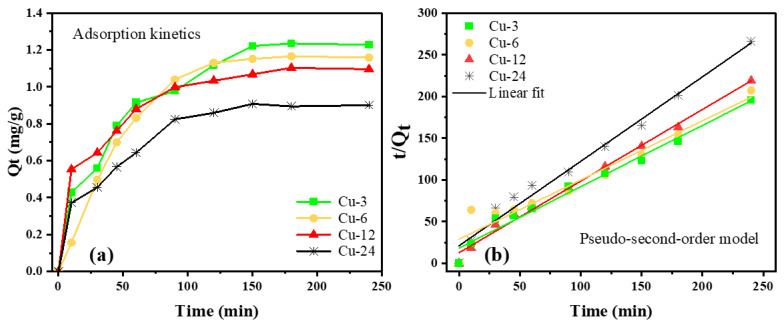
Adsorption kinetics (**a**) and the pseudo-second-order kinetic (**b**) plots for 5 mg of the nanocomposite in 15 mL aqueous solution containing a 5 mg/L concentration of MB.

Adsorption kinetics were studied at different time intervals in order to quantify the adsorption rate and elucidate the mechanism of adsorption, i.e., physical or chemical adsorption. Adsorption kinetics describe the rate of release of a sorbate from an aqueous solution to a solid-phase interface. The isotherms of MB adsorbed on the surface of the different nanoparticle mixture samples are show in Figure 6a. In dark conditions, the MB adsorbed on the surface of the nanoparticles exhibits an abrupt increase up to the first 100 min after which it reaches a plateau. This initial uptake is due to the abundance of active sites on the surface of the nanoparticles, which decreases over time, i.e., when solute concentration gradient becomes very high. When C_e_/C_0_ reaches a plateau, it suggests that all active sites have been filled and no additional adsorption is possible. Furthermore, the adsorption–desorption equilibrium is achieved after ~2 h. This trend indicates the layer-by-layer (monolayer to multi-layer) adsorption of MB on the surface of the Cu-based nanoparticles [57]. The MB removal for the Cu-3, Cu-6, Cu-12 and Cu-24 samples is 8.04%, 7.58%, 7.16% and 5.77%, respectively, after 250 min. Additionally, Q_t_ is higher for samples containing mixed phases of Cu, Cu_2_O and Cu_3_N and lower for the pure Cu phase nanoparticles.

For further investigation, the pseudo-second-order model was also applied in order to interpret the adsorption kinetics (Figure 6b). In the pseudo-second-order model, we assume that (i) the adsorbate concentration is constant in time and (ii) the total number of binding sites depends on the amount adsorbed at equilibrium. Furthermore, this model is most adapted to chemical sorption involving valence forces through sharing or exchanging of electrons between adsorbent and sorbate, as in the case of organic pollutants. A linear relationship with high correlation coefficients (R^2^) is observed between t/Q_t_ and t, indicating the applicability of the pseudo-second-order model to describe the adsorption process of MB with the nanoparticle mixtures. In Table 2, the values of the correlation coefficient R^2^ suggest that the adsorption tendency corroborates with the pseudo-second-order kinetic model with a slight improvement for samples containing larger amounts of Cu nanoparticles (Cu-12 and Cu-24). The amount of MB adsorbed per gram of the photocatalyst (Q_e_) is the highest for the Cu-3 and Cu-6 samples. The rate constant K_2_ for pseudo-second-order was the lowest for the Cu-6 sample followed by the Cu-3 sample, indicating quicker reaction kinetics for these samples containing higher amounts of Cu_3_N and Cu_2_O phases. In fact, the co-precipitation of the Cu phase during synthesis indicates that both Cu_2_O and Cu_3_N were synthesized in Cu-rich conditions, likely resulting in oxygen and nitrogen vacancies on their surface. These positively charged defects are active areas for chemical adsorption and improved absorption kinetics.

## 5. Sunlight-Driven Photocatalysis

**Figure 7:**
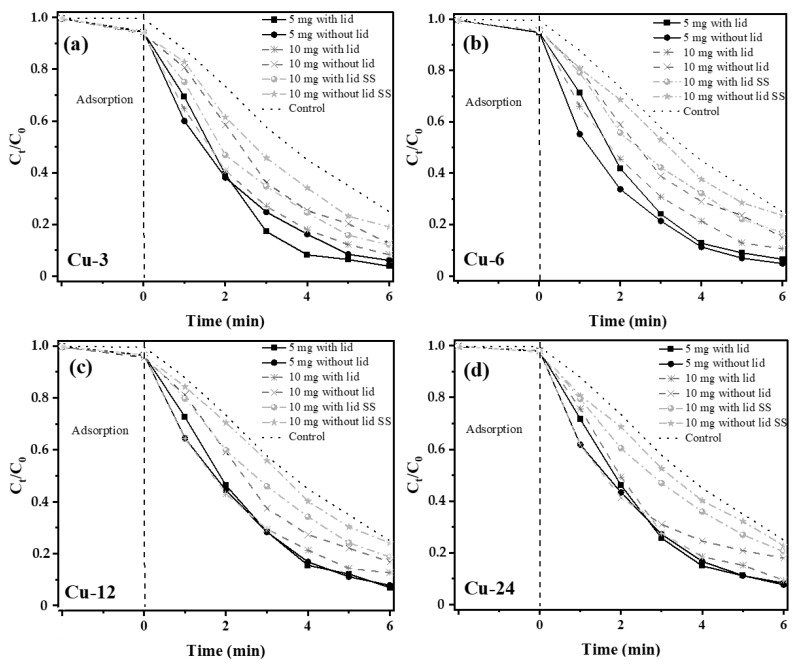
Normalized MB degradation curves under sunlight radiation with (**a**) Cu-3, (**b**) Cu-6, (**c**) Cu-12 and (**d**) Cu-24 in 20 mL aqueous solution with an MB concentration of 5 mg/L.

Visible light photocatalytic activity of the nanoparticle mixture samples was subsequently evaluated by measuring the removal or degradation of MB from an aqueous solution under sunlight radiation with a UV index of 4–5. The photodegradation of MB was monitored with the normalized change in its concentration (C_t_/C_0_) as a function of the irradiation time (t), as shown in Figure 7. All the solutions tested consisted of 5 mg/L of MB in distilled water except for the control sample, which did not contain any nanoparticles. Under solar radiation, the photobleaching in the control sample is observed. However, the tests with nanoparticles showed that the photocatalytic degradation of MB is higher than the photobleaching in the control sample and reaches 55% degradation after 4 h of sunlight exposure. Furthermore, an equilibrium is achieved after 6 h, and the photocatalytic degradation rate decreases. MB degradation of 96%, 95%, 93% and 92% using the Cu-3, Cu-6, Cu-12 and Cu-24 samples, respectively, is obtained using 5 mg of nanocomposites for each experiment. All experiments were carried out in beakers without a lid. The higher degradation efficiency observed for the Cu-3 sample can be ascribed to the augmented visible light absorption range, owing to a higher amount of Cu_3_N nanoparticles in the sample. The presence of these phases broadens the absorption spectra up to the infrared. In addition, the high specific surface of the Cu_3_N and Cu_2_O nanoparticles is directly linked to their average particle sizes of 2.9 nm and 15 nm, respectively. As the synthesis time increases, the particles enlarge, and as a consequence, the specific surface is lowered. In addition, the SPR of Cu nanoparticles would create charge polarization that can influence the absorption and scattering of visible light, in turn, enhancing the photocatalytic degradation efficiency of Cu_3_N and Cu_2_O [58].

The photocatalytic degradation efficiency depends on the active surface area of the photocatalyst. For higher amounts of catalysts, i.e., 10 mg, the dye-degradation efficiency decreases to 87%, 85%, 83% and 82% for the Cu-3, Cu-6, Cu-12 and Cu-24 samples, respectively, at the end of 6 h. It has been reported that the photocatalytic degradation efficiency increases as the amount of photocatalyst increases up to an optimum quantity of catalyst, after which a decrease in degradation is observed [59]. In this particular study, the nanocomposites are hydrophobic and remain on the surface of the aqueous solution. For 5 mg of catalyst, a thin layer of nanoparticles is formed on the surface of the liquid, which thickens with an increase in the catalyst amount, making the surface opaque. Additionally, only the topmost layers in the catalyst surface are exposed to sunlight, which may not be in contact with the aqueous solution. This implies that the charge transfer from the nanoparticles to the organic pollutants in the aqueous solution may be less efficient. This shielding effect due to the increase in the quantity of the photocatalyst could explain the lower photocatalytic activity. In addition, at high concentrations, the photocatalyst nanoparticles have a tendency to aggregate, which, in turn, reduces the number of active sites. Khataee et al. reported similar results, in which degradation efficiency increases from 14% to 57% with a catalyst dosage from 0.25 to 1 g/L, followed by a decrease in the degradation efficiency by 50% with a further increase in the catalyst dosage up to 2 g/L [60]. In fact, for very high concentrations of catalysts, the increase in turbidity of the suspension decreases the light penetration and the photodegradation efficiency [61].

The photocatalytic dye degradation in several Cu-based nanomaterials is presented in Table 3, for comparison. In general, their dye degradation efficiency is very high, depending upon the dye, excitation source and catalyst load. However, most of the photocatalytic degradation activity is carried out under high-power UV- light, which showed degradation efficiencies of up to 90% for various organic dyes. Most of the studies used higher catalyst amounts owing to higher C_0_. Under sunlight or solar simulator, the degradation efficiencies were lower ranging between 65 and 80%. In comparison, the nanoparticle mixtures in this study manifest very high degradation efficiencies under sunlight, reaching 96% for Cu-3, consisting of the highest quantity of Cu_3_N nanoparticles.

In this section, the effect of blocking UV-radiation, in order to exclusively study visible solar-driven photocatalytic degradation of MB, is analyzed. The absorption spectra of the nanoparticle mixtures provide an additional peak at ~300 nm (Figure S3), which corresponds to the absorption peak of oleylamine, corroborating our FTIR studies [64]. The presence of this organic moiety shields the nanoparticle surface and could have a negative effect on their photocatalytic efficiencies. For this, a glass lid is placed on the beakers during sunlight exposure. Figure S5 provides the transmittance characteristics of distilled water (DI), MB solution and the glass cover/lid. As observed, DI transmits from the UV to infrared with an absorption peak at around 980 nm. The transmittance of DI with MB is very similar to the transmittance of DI alone, except for a strong absorption peak at 660 nm corresponding to MB absorption. However, the glass lid transmits uniformly from 400 nm to 1100 nm, i.e., from the visible to the infrared regions. The purpose of the lid was therefore to limit the photoexcitation wavelengths from 400 nm to 1100 nm, allowing us to study MB degradation under visible light. For the 5 mg catalyst loading, the lid did not change the photocatalytic degradation efficiency significantly. On the other hand, for the 10 mg loading, an overall reduction in the photocatalytic efficiency is observed compared to the 5 mg loading, irrespective of the lid. Considering the 10 mg loading with and without the lid, the presence of the lid improves the degradation efficiency by 10% with MB degradations of 92%, 89%, 88% and 90% for the Cu-3, Cu-6, Cu-12 and Cu-24 samples, respectively. In order to reduce the shielding effect mentioned before, the 10 mg catalyst-loaded MB solution was sonicated (represented as SS on the graphs in Figure 7) in order to homogeneously disperse the nanoparticles in the solution. After sonication, the turbidity of the solution increased, which consequently decreased the photocatalytic degradation by 10% compared to the non-sonicated samples. In fact, the MB degradation with the lid was 83%, 88%, 81% and 79%, and without the lid, it was 76%, 81%, 76% and 77%, for the Cu-3, Cu-6, Cu-12 and Cu-24 samples, respectively. Since, both Cu_2_O and Cu_3_N are p-type semiconductors, when in contact with metallic Cu, air or water, they undergo downward band bending, creating an energy barrier for the photogenerated holes [65]. Band bending is an important mechanism for tuning the surface properties of a semiconductor. For p-type semiconductors in contact with O-rich environments, such as in aqueous media, the conduction band and valence band undergo downward band bending. This phenomenon tends to reduce the excitonic radiative recombination in the band-bending region [66]. In nanomaterials with a high surface-to-volume ratio, it results in an overall decrease in radiative recombination. In such situations, excitonic relaxation occurs via non-radiative pathways. Furthermore, downward band bending results in electron accumulation near the surface of the nanoparticles on photoexcitation. Subsequently, the transfer of electrons to oxygen molecules and hydroxyl radicals in contact with the nanoparticle surface occurs in aqueous media via a type II mechanism of dye degradation. Thereupon, oxidizing species, such as singlet oxygen, superoxide, hydroxyl radical and ion, are generated that degrade organic pollutants, such as MB, as shown in Figure 5b. Even though, dyes undergo photobleaching under the appropriate radiation via type I and II mechanisms, the presence of a semiconductor photocatalyst produces a high number of electrons that are transferred to the oxygen molecules that subsequently create a higher amount of ROS, which, in turn, enhances the dye degradation. After several oxidations of the oxygen molecule to either the hydroxyl radical or ions that degrade the dye, they finally condense into water, which is the final product of the photocatalytic process. Even though Cu nanoparticles themselves demonstrate good photocatalytic efficiency via efficient production of ROS, the samples with Cu_3_N tend to possess a higher catalytic efficiency of ~4%. However, the catalytic efficiency of Cu nanoparticles decreases with higher catalyst loading. Additionally, samples containing Cu_3_N tend to present higher adsorption kinetics than the Cu-24 sample containing only Cu nanoparticles. Therefore, the combined effect of adsorption and photocatalysis is required in order to obtain a higher dye-degradation efficiency.

## 6. Conclusions

In summary, we have successfully synthesized Cu-based nanocomposites with different ratios of Cu, Cu_2_O and Cu_3_N using non-aqueous sol-gel routes by varying the synthesis time. These were successfully applied to the visible-light-driven photocatalytic degradation of MB. The presence of the plasmonic Cu metal nanoparticles in these samples induces downward band bending in the p-type Cu_2_O and Cu_3_N, creating electron accumulation on their surfaces. Therefore, the mechanism of dye degradation in addition to photobleaching is the generation of reactive oxygen species on the transfer of electrons from the nanoparticle surface to the oxygen and hydroxyl radicals present in the aqueous medium. In narrow band gap semiconductors, the rapid excitonic recombination rate that tends to decrease the photocatalytic efficiency is circumvented via the band bending effect in the p-type semiconductor at the metal interface as well as at the aqueous interface. Other parameters that enhance the photocatalytic activity include the increase in the absorption range of the photocatalyst, their morphology, higher specific surface, optimum catalyst dosage and the homogeneity of the catalyst dispersion in the solution. This study showed that the presence of Cu_3_N extends the light absorption range to the near-infrared region, with an effective absorption range from 600 nm to 1000 nm for the nanoparticle mixtures adapted to visible light photocatalysis. The photocatalytic degradation is also the highest for the sample containing nanoparticles with the highest specific surface, i.e., Cu_2_O and Cu_3_N. However, the stability of these nanoparticles still needs to be explored as Cu_3_N and Cu can undergo oxidation and Cu_2_O can further oxidize to CuO. Another challenge remains in applying these nanomaterials to larger-scale setups and reclaiming the catalysts after exhaustion. Therefore, our future work will consist of understanding the stability of these nanocomposites for various cycles of dye degradation by immobilizing them on appropriate supports to facilitate their recovery.

## Figures and Tables

**Table 1:** Phase ratios of Cu_2_O, Cu_3_N and Cu in the samples and their crystallite size distribution calculated from the PXRD patterns.

	Cu-3		Cu-6		Cu-12		Cu-24	
Crystalline Phase	Crystallite Size (nm)	Crystalline Phase (%)	Crystallite Size (nm)	Crystalline Phase (%)	Crystallite Size (nm)	Crystalline Phase (%)	Crystallite Size (nm)	Crystalline Phase (%)
Cu	50	32.1	55.7	44.6	54	90.3	71	100
Cu_2_O	14.8	49.5	26.7	54.3	30	5.5	-	-
Cu_3_N	2.8	18	30	1.1	29	4.2	-	-

**Table 2:** Adsorption kinetic parameters of MB by the nanocomposites obtained using the pseudo-second-order kinetic Equation (3).

Sample	Pseudo-Second-Order		
	Q_e,fitted_ (mg/g)	K_2_ (g/mg min)	R^2^
Cu-3	1.3089	0.0296	0.9783
Cu-6	1.4080	0.0174	0.9361
Cu-12	1.1629	0.0588	0.9821
Cu-24	0.9862	0.0431	0.9920

**Table 3:** Comparison of photocatalytic performance of recent works using Cu-based nanomaterials and the present study.

Catalyst	Dye	Light Source	Initial Concentration	Catalyst Dosage	Degradation Rate (%)	Ref.
CuO-Cu_2_O	MB Methylene Orange	150 W metal halide lamp	5 mg/L	0.2 g	80	[8]
					50	
CuO	Aniline	UV-C lamp	50 mg/L	0.01–0.1 g	90	[12]
Cu_2_O	Fluroxypyr	500 W metal halide lamp	11.17 mg/L	0.1 g	83	[13]
Cu_3_N	MB	Solar simulator	20 mg/L	0.1 g	61	[14]
	Methylene Orange				89	
Au-Cu_3_N	MB	250 W UV lamp	25 mg/L	15 mg	84	[25]
Cu_2_O/CuO/Cu	MB	Sun light	20 mg/L	40 mg	65	[62]
	Rhodamine B				60	
Cu-ZnO	Methylene Orange	30 W UV-light lamp	10 mg/L	25 mg	91	[63]
	Indigo Carmine				92	
	Rhodamine B				90	
Cu-3	MB	Sun light	5 mg/L	5 mg	96	Present study
Cu-6					95	
Cu-12					93	
Cu-24					92	

## Data Availability

Not applicable.

## Supplementary Materials

The following supporting information can be downloaded at: https://www.mdpi.com/article/10.3390/nano13081311/s1, Figure S1: Calibration curve of methylene blue with the Lovibond photometer; Figure S2: EDX of the truncated octahedron in sample 6 h; Figure S3: UV-Vis absorption spectrum of the 6 h sample with an absorption peak at ~300 nm; Figure S4: Tauc plot of Cu-3. Figure S5: UV-Vis spectra of distilled water (DI), methylene blue (MB) and the glass cover.
