# Supplementary material for: Sunlight-Driven Photocatalytic Degradation of Methylene Blue with Facile One-Step Synthesized Cu-Cu2O-Cu3N Nanoparticle Mixtures
Source: Nanomaterials (Basel). 2023 Apr 8;13(8):1311. doi: 10.3390/nano13081311 (PMC10144494; doi:10.3390/nano13081311)
Supplement: Supplementary file 1 [file nanomaterials-13-01311-s001.zip › nanomaterials-2329532-supplementary.pdf]

nanomaterials

Supporting Information

Sunlight-Driven Photocatalytic Degradation of Methylene Blue with Facile One-Step Synthesized Cu-Cu_2O-Cu_3N Nanoparticle Mixtures

Patricio Paredes [1], Erwan Rauwel [1], David S Wragg [2], Laetitia Rapenne [3], Elias Estephan [4], Olga Volobujeva [5] and Protima Rauwel [1,*]

[1] Institute of Forestry and Engineering Sciences, Estonian University of Life Sciences, Kreutzwaldi 56/1, 51014 Tartu, Estonia;
[2] Department of Chemistry and SMN, University of Oslo, 0315 Oslo, Norway;
[3] Grenoble Institute of Engineering, LMGP, University Grenoble Alpes, CNRS, F-38000 Grenoble, France;
[4] Laboratory of Bioengineering and Biosciences, LBN, Univ Montpellier, 34193 Montpel-lier , France;
[5] Institute of Materials and Environmental Technology, Tallinn University of Technology, 19086 Tallinn, Estonia;
* Correspondence: protima.rauwel@emu.ee

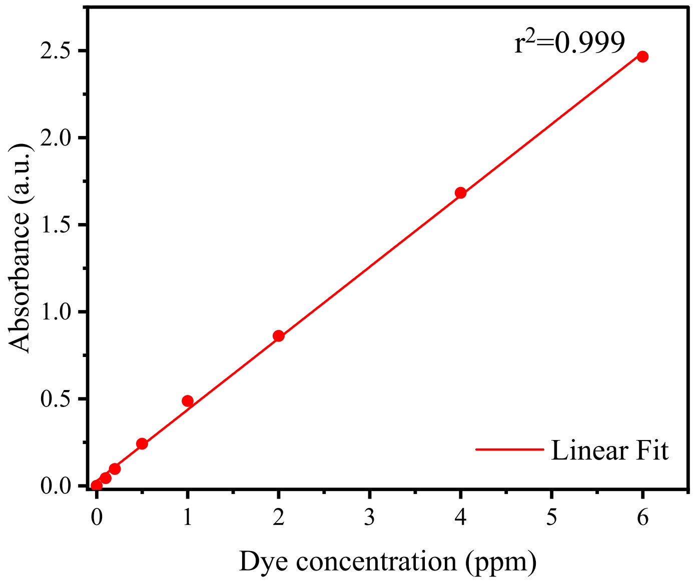

Figure S1. Calibration curve of MB using Lambert-Beer's law obtained with Lovibond photometer at a wavelength of 660nm.

For the calibration curve, eight samples at different concentrations of 6, 4, 2, 1, 0.5, 0.2, 0.1 and 0 mg/L of MB were prepared. The absorbance of the solutions was measured using a Lovibond photometer with a wavelength of 660nm and plotted as a function of MB concentration.

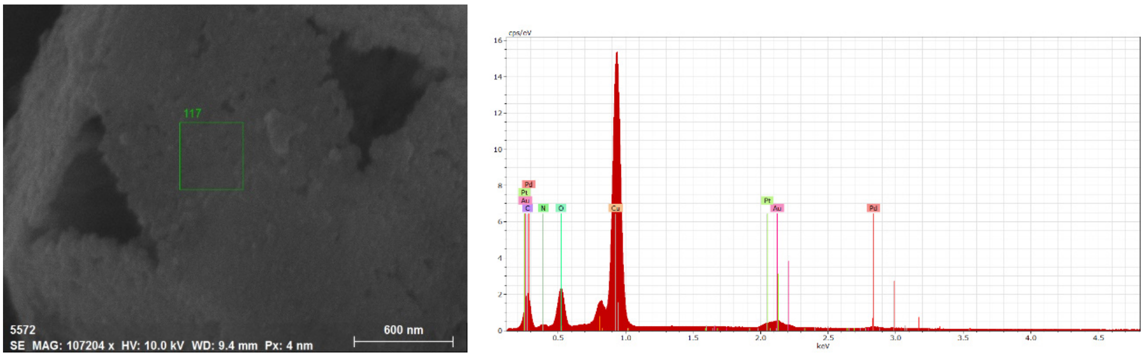

Element	Series	unn. C [wt.%]	norm. C [wt.%]	Atom. C [at.%]	Error (1 Sigma) [wt.%]
Copper	L-series	89.88	89.88	68.66	9.52
Oxygen	K-series	8.66	8.66	26.26	1.04
Nitrogen	K-series	1.46	1.46	5.08	0.26
	Total:	100.00	100.00	100.00	

Figure S2: EDX of the truncated octahedron in sample 6h.

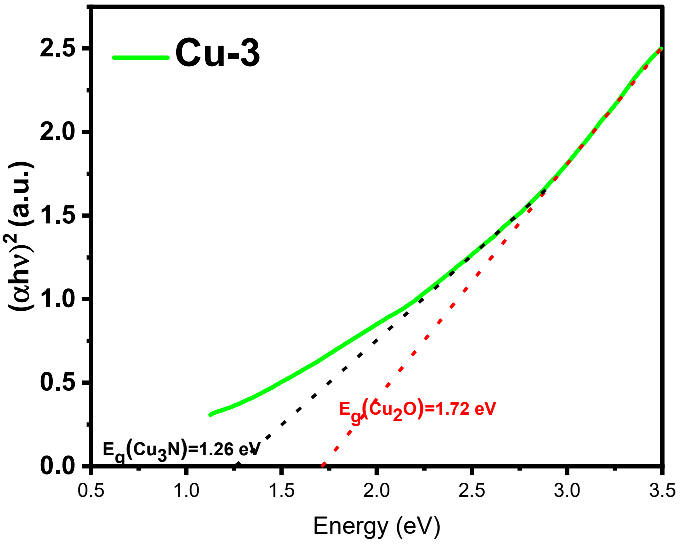

Figure S3. Tauc plots of Cu-3 obtained from UV-Vis absorption spectrum of the sample. The extrapolation of the linear portions of the curves provide the bang gap of the Cu_3N and Cu_2O phases.

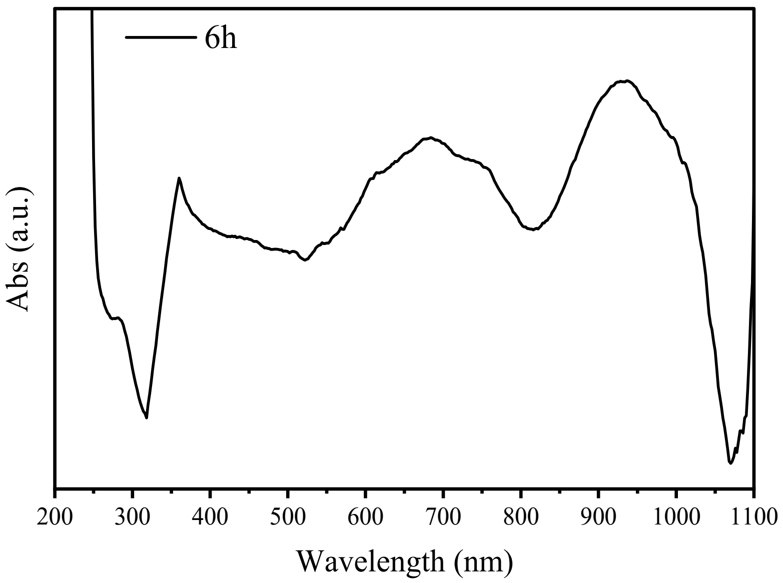

Figure S4. UV-Vis absorption spectrum of 6 h sample showing the absorption peak at ~300 nm.

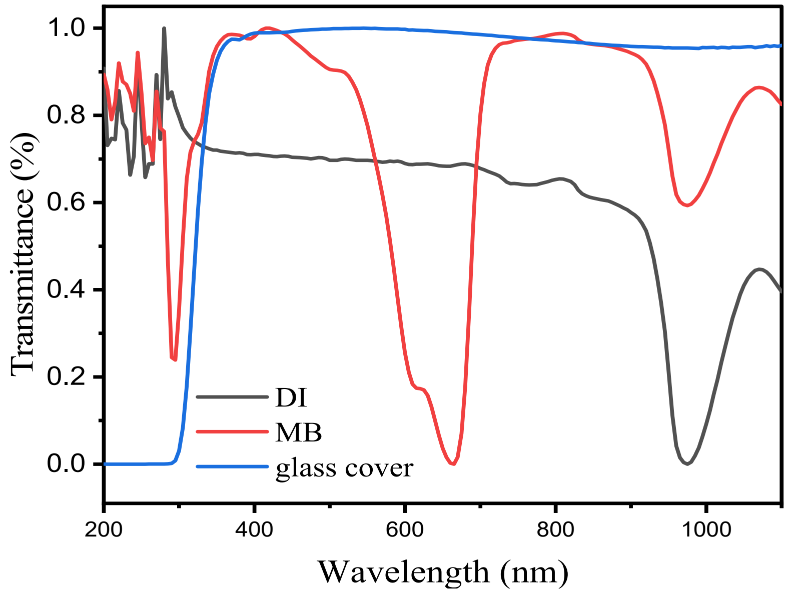

Figure S5. S. UV-Vis spectra of distilled water (DI), Methylene Blue (MB) and glass cover.
